# Bacterial genome-wide association studies: exploring the genetic variation underlying bacterial phenotypes

**DOI:** 10.1128/aem.02512-24

**Published:** 2025-05-16

**Authors:** Qiuping Yang, Xiaoqi Wang, Mengting Han, Huanjing Sheng, Yulu Sun, Li Su, Wenjing Lu, Mei Li, Siyue Wang, Jia Chen, Shenghui Cui, Bao-Wei Yang

**Affiliations:** 1 College of Food Science and Engineering, Northwest A&F University 538750, Shaanxi, China; 2 College of Chemical Technology, Shijiazhuang University 71181, Shijiazhuang, China; 3 National Institutes for Food and Drug Control 12540, Beijing, China; University of Illinois Urbana-Champaign, Urbana, Illinois, USA

**Keywords:** bacterial GWAS, bacterial phenotypes, BGWAS workflow, bioinformatics tools, confounding factors

## Abstract

With the continuous advancements in high-throughput genome sequencing technologies and the development of innovative bioinformatics tools, bacterial genome-wide association studies (BGWAS) have emerged as a transformative approach for investigating the genetic variations underlying diverse bacterial phenotypes at the population genome level. This review provides a comprehensive overview of the application of BGWAS in elucidating genetic determinants of bacterial drug resistance, pathogenicity, host specificity, biofilm formation, and probiotic fermentation characteristics. We systematically summarize the BGWAS workflow, including study design, data analysis pipelines, and the bioinformatics software employed at various stages. Furthermore, we highlight specialized tools tailored for BGWAS and discuss their unique features and applications. We also discuss confounding factors that can influence the accuracy and reliability of BGWAS results, including population structure, linkage disequilibrium, and multiple testing. By incorporating recent advancements, this review serves as a comprehensive reference for researchers utilizing BGWAS to uncover the genetic basis of bacterial phenotypes.

## INTRODUCTION

Genome-wide association studies (GWAS) involve identifying genetic polymorphisms across the genomes of multiple individuals. Subsequently, these genetic variants and their corresponding phenotypes are statistically analyzed at the population level to identify loci that potentially drive phenotypic variation (1, 2). The concept of GWAS originated in the 1990s and was first proposed by Risch and Merikangas in their research on the genetics of complex diseases (3). In 2005, *Science* magazine published the first GWAS associated with age-related macular degeneration, signifying the emergence of GWAS analysis (4). Since then, GWAS has become an indispensable tool in genetic disease study, offering essential insights into the genetic mechanisms of complex diseases and facilitating drug development. It has been used to identify genetic variants associated with diseases such as coronary artery disease, type 2 diabetes, Alzheimer’s disease, and lung cancer (5–8). Furthermore, GWAS has been extensively employed to elucidate the genetic mechanisms underlying various phenotypes in both animals and plants. Previous studies have demonstrated that GWAS has elucidated genetic variants influencing chicken breast production (9), meat quality traits in chicken (10), livability and six disease traits in Holstein cattle (11), soybean aphid resistance in soybean (12), drought tolerance in Iranian bread wheat (13), and yield-related traits in soft red winter wheat (14). Overall, GWAS represent a powerful approach for identifying genetic variants associated with complex traits (15).

Bacteria exhibit remarkable genetic and phenotypic diversity, driven by mutation, horizontal gene transfer, and selective pressures from various environmental and host-associated factors. Moreover, bacterial cells with identical genotypes frequently display different phenotypes, highlighting the complexity of genotype–phenotype relationships. However, traditional genetic approaches often struggle to resolve these intricate associations due to the multifactorial nature of bacterial traits. To address this challenge, GWAS has emerged as a powerful tool for systematically linking genetic variations to phenotypic traits across bacterial populations. In 2013, Sheppard et al. (16) pioneered the application of GWAS in *Campylobacter*, demonstrating vitamin B5 biosynthesis as a host specificity factor in this organism. Since then, an increasing number of studies have employed GWAS to investigate the genetic characteristics of various bacteria, such as *Escherichia coli* (17), *Streptococcus pneumoniae* (18), *Neisseria gonorrhoeae* (19), *Mycobacterium tuberculosis* (20), *Staphylococcus aureus* (21), and lactic acid bacteria (LABs) (22). As bacterial GWAS continue to expand, they hold great promise for elucidating the genetic determinants of clinically and ecologically significant traits, offering new avenues for antimicrobial resistance surveillance, vaccine development, and precision microbiology.

Due to the unique characteristics of bacterial reproduction, bacterial genomes tend to be shaped by stronger positive selection, stronger linkage disequilibrium (LD), and stronger population stratification than humans, with implications for GWAS power and resolution (23). Furthermore, there is currently no standardized protocol for bacterial genome-wide association studies (BGWAS), especially regarding the number of strains required, types of genetic variants considered, and choice of analytical tools. Given these complexities, this review aims to (i) summarize the applications of GWAS in investigating various bacterial phenotypes (e.g., drug resistance, pathogenicity, host specificity, biofilm formation, and probiotic fermentation characteristics); (ii) compare commonly used BGWAS software tools; and (iii) discuss potential confounding factors that may influence the accuracy and interpretability of BGWAS results.

## APPLICATIONS OF GWAS IN BACTERIA

The genetic variants underlying reproducible and quantifiable bacterial phenotypes (e.g., drug resistance, growth plasticity, pathogenicity, host specificity, and biofilm formation) can be effectively investigated through BGWAS (16, 21, 24–27) (Table S1; Table 1).

**TABLE 1:** Summary of BGWAS in published literature^*a*^

Species	Phenotypes	Genetic variants	BGWAS tools
*Acinetobacter baumannii*	Drug resistance (FEP, CXM, GEN, CAZ, TMP, AZM, CRO, ATM, ERY, PIP, LVX, IMP, CIP, CXM-AXT, CFZ, CTT, SAM, CFP-SUL, AMP, TOB, SXT, NIT, and TGC) and transformation rates	SNPs, k-mers, and unitigs	Bugwas, GEMMA, treeWAS, Pyseer, and A custom Python script
*Bacillus cereus*	Isolate sources, motility, oxygen requirement, cereulide production levels, and pathogenicity (anthrax or food poisoning)	Genes and unitigs	Scoary and Pyseer
*Campylobacter jejuni*	Pathogenicity and biofilm formation	k-mers, SNPs and genes	phyC and μPathML
*Clostridioides difficile*	Cytotoxicity; Ribotypes; Drug resistance (MET, MXF, RIF, VAN)	SNPs, indels, genes, and unitigs	Hogwash, treeWAS, and DBGWAS
*Cutibacterium acnes*	Pathogenicity	Genes	Scoary
*Enterococcus* spp.	Pathogenicity; drug resistance (CHL, VAN, CLI, ERY, D/Q, RIF, and KAN); clade membership; and biofilm robustness	SNPs, genes, and unitigs	Scoary, Pyseer, FastLmmC, and treeWAS
*Escherichia coli*	Drug resistance (NAL, NOR, CIP, LVX, AMP, CHX, ATM, CAZ, CFZ, CTT, CRO, SAM, DOX, GEN, SXT, and MXF); pathogenicity; migration rates; host specificity; conjugation efficiency; biofilm formation; growth interactions; isolate sources; and microcin resistance (microcin C, microcin J25, microcin B17, and microcin E492)	SNPs, k-mers, unitigs, and genes	Scoary, treeWAS, Pyseer, HAWK, Q-ROADTRIPS, and Bugwas
*Helicobacter pylori*	Pathogenicity	SNPs, k-mers, and unitigs	Pyseer and Bugwas
*Klebsiella pneumoniae*	Drug resistance (ETP, MEM, IMP, CFZ, CXM, CRO, CIP, GEN, and TOB); pathogenicity; clone specificity; and host specificity	SNPs, genes, and k-mers	Scoary, treeWAS, Pyseer, and Bugwas
*Lactobacillus* spp.	Carbohydrate metabolism; fermentation (curding time, fermentation time, acid production rate, and protein hydrolysis rate); niche specificity; and pathogenicity (health vs bacterial vaginosis)	Genes and SNPs	Scoary and treeWAS
*Lactococcus* spp.	Nitrogen fixation and fermentation capability (curding time, fermentation end time, acidification rate, proteolytic ability, and other phenotypic indicators)	Genes and SNPs	Scoary and treeWAS
*Listeria monocytogenes*	Biofilm formation; pathogenicity; growth at low temperature; organic acid tolerance (acetic, lactic and propionic acids); adhesion ability; biocide resistance (BC, DDAC, PHMB, EtOH, PA, HP, HS, and AMPD); persistence on farms; isolate sources; and survival rate in soil	Genes, unitigs, SNPs, and k-mers	Pyseer, treeWAS, Bugwas, Scoary, and GEMMA
*Mycobacterium tuberculosis*	Drug resistance (AMC, AMK, BDQ, CAP, CIP, DCS, DLM, EMB, ETA, INH, KAN, LVX, LIN, MXF, OFL, PAS, PZA, RFB, RIF, and STM); pathogenicity; transmissibility; isolate sources; growth assay; and prevalence phenotype (high and low prevalence traits)	SNPs or indels, k-mers, unitigs, and genes	GEMMA, PhyC, Scoary, PLINK, Hogwash, Pyseer, Bugwas, treeWAS, Scoary, DBGWAS, and A custom Python script
*Neisseria gonorrhoeae*	Drug resistance (AZM, PEN, and TET) and CO_2_ dependence	Unitigs and k-mers	Pyseer
*Neisseria meningitidis*	Pathogenicity, drug resistance (PEN), and isolate sources		
*Pseudomonas aeruginosa*	Biofilm formation; pathogenicity; drug resistance (AMK, FEP, CAZ, CIP, CST, IMP, MEM, PIP, TZP, and TOB); and murepavadin resistance	SNPs and indels, k-mers, and genes	Scoary, PLINK, and Pyseer
*Salmonella*	Drug resistance (CST, AMP, CHL, CIP, SXT, CTX, NAL, AMC, NIT, TET, and CAZ); host specificity; and biofilm formation	SNPs and indels and genes	GEMMA, Scoary, and Pyseer
*Staphylococcus aureus*	Growth plasticity; drug resistance (DAP, CPT, VAN, CIP, ERY, FA, GEN, PEN, MET, TET, TMP, and OXA); pathogenicity; delta-toxin levels; host specificity; capsule production; phage sensitivity; clade specificity; cytotoxicity phenotype; and isolate sources	SNPs, unitigs, genes, and k-mers	GEMMA, PLINK, SEER, Scoary, treeWAS, Bugwas, DBGWAS, Pyseer, and FastLmmC
*Streptococcus pneumoniae*	Pathogenicity; drug resistance (PEN, ERY, TMP, SXT, CIP, and OFL); and clade specificity	SNPs and indels, genes, k-mers, and unitigs	Scoary, PLINK, Pyseer, FaST-LMM, GEMMA, and SEER
*Vibrio* spp.	Host specificity; pathogenicity; drug resistance under anaerobic and aerobic conditions (CIP, AZM, and DOX); and isolate sources	SNPs, k-mer, genes, and unitigs	Pyseer, DBGWAS, Scoary, and PLINK

^*a*^ AMC, amoxicillin-clavulanate; AMK, amikacin; AMP, ampicillin; AMPD, N-(3-aminopropyl)-N-dodecylpropane-1 3-diamine; ATM, aztreonam; AZM, azithromycin; BC, benzalkonium chloride; BDQ, bedaquiline; CAP, capreomycin; CAZ, ceftazidime; CFP-SUL, cefoperazone-sulbactam; CFZ, cefazolin; CHL, chloramphenicol; CHX, chlorhexidine; CIP, ciprofloxacin; CLI, clindamycin; CPT, ceftaroline; CRO, ceftriaxone; CST, colistin; CTT, cefotetan; CTX, cefotaxime; CXM, cefuroxime; CXM-AXT, cefuroxime axetil; DAP, daptomycin; DCS, D-cycloserine; DDAC, didecyl dimethyl ammonium chloride; DLM, delamanid; DOX, doxycycline; D/Q, quinupristin-dalfopristin; EMB, ethambutol; ERY, erythromycin; ETA, ethionamide; EtOH, ethanol; ETP, ertapenem; FA, fusidic acid; FEP, cefepime; GEN, gentamicin; HP, hydrogen peroxide; HS, sodium hypochlorite; IMP, imipenem; indels, insertions and deletions; INH, isoniazid; KAN, kanamycin; LIN, linezolid; LVX, levofloxacin; MEM, meropenem; MET, methicillin; MXF, moxifloxacin; NAL, nalidixic acid; NIT, nitrofurantoin; NOR, norfloxacin; OFL, ofloxacin; OXA, oxacillin; PAS, para-aminosalicylic acid; PEN, penicillin; PIP, piperacillin; PZA, pyrazinamide; RFB, rifabutin; RIF, rifampicin; SAM, ampicillin-sulbactam; SNPs, single nucleotide polymorphisms; STM, streptomycin; SXT, trimethoprim-sulfamethoxazole; TET, tetracycline; TGC, tigecycline; TMP, trimethoprim; TOB, tobramycin; TZP, piperacillin-tazobactam; VAN, vancomycin; PA, peroxyacetic acid; PHMB, polyhexamethylene biguanide.

### GWAS and bacterial drug resistance

Bacterial antibiotic resistance complicates infection management, reduces therapeutic efficacy, increases treatment costs, and poses a significant threat to global public health. In 2019, the World Health Organization identified antibiotic resistance as one of the top 10 threats to global health. A study published in *The Lancet* estimated that 4.95 million deaths were associated with antibiotic resistance in 2019, of which 1.72 million deaths were directly attributable to it (28). Given the severity of antibiotic resistance, effective control measures are urgently needed. A comprehensive understanding of the mechanisms underlying bacterial resistance is crucial for reducing and controlling resistance. Fortunately, GWAS have emerged as a powerful tool to identify genetic variants associated with antibiotic resistance. By analyzing large data sets of bacterial genomes, GWAS can provide valuable insights into the resistance mechanisms and facilitate the discovery of new drug targets.

BGWAS have been extensively utilized in investigating the genetic determinants of drug resistance across various clinically relevant pathogens, such as penicillins (ampicillin [AMP] and piperacillin), cephalosporins (cefazolin [CFZ], cefuroxime [CXM], cefotetan [CTT], ceftazidime [CAZ], ceftriaxone [CRO], and cefepime), carbapenems (imipenem [IMP]), aminoglycosides (gentamicin [GEN] and tobramycin [TOB]), quinolones (levofloxacin [LVX] and ciprofloxacin [CIP]), macrolides (azithromycin and erythromycin), sulfonamides (trimethoprim and trimethoprim-sulfamethoxazole [SXT]), aztreonam, nitrofurantoin, and tigecycline in *Acinetobacter baumannii* (29, 30); penicillins (AMP), cephalosporins (CFZ, CTT, CAZ, CRO, and cefotaxime), quinolones (nalidixic acid, norfloxacin, CIP, LVX, and moxifloxacin [MXF]), aminoglycosides (GEN), tetracyclines (doxycycline), sulfonamides (SXT), and chlorhexidine in *Escherichia coli* (31–34); cephalosporins (CFZ, CXM, and CRO), carbapenems (IMP, ertapenem, and meropenem), quinolones (CIP), and aminoglycosides (GEN and TOB) in *Klebsiella pneumoniae* (31, 35, 36); and aminoglycosides (amikacin, kanamycin, and streptomycin), quinolones (CIP, LVX, MXF, and ofloxacin), rifamycins (rifabutin and rifampicin), and antituberculosis agents (bedaquiline, delamanid, pyrazinamide, isoniazid, para-aminosalicylic acid, clofazimine, D-cycloserine, ethambutol, ethionamide, and linezolid) in *Mycobacterium tuberculosis* (20, 37–40) (Table 1; Table S1).

BGWAS have identified novel loci associated with drug resistance. GWAS conducted on *Klebsiella pneumoniae* revealed genetic variants linked to carbapenems resistance, identifying 28 and 37 potential marker genes associated with imipenem and meropenem resistance, respectively. These genes were likely related to biofilm formation, efflux pump, and DNA repairing (41). In *Staphylococcus aureus*, GWAS revealed MprF P314L and L826F to be significantly associated with daptomycin resistance (21). Mutations in *bdc*A (involved in biofilm dispersal) and *val*S (encoding an aminoacyl-tRNA synthetase) genes in *Escherichia coli* have been implicated in quinolone resistance (32). These findings underscore the utility of BGWAS in uncovering novel genetic determinants of drug resistance, many of which may not have been identified through conventional genetic approaches. However, while GWAS can reveal statistically significant associations, functional validation through wet-lab experiments remains crucial to confirming their biological relevance. It can provide direct evidence of how these genetic variants contribute to resistance mechanisms. Furthermore, integrating GWAS findings with laboratory validation not only enhances our understanding of bacterial physiology and resistance evolution but also helps mitigate potential false positives inherent in association studies. As BGWAS continues to expand, its integration with experimental validation will be essential for accurately identifying and characterizing resistance determinants, ultimately informing antimicrobial stewardship strategies and novel therapeutic targets.

### GWAS and bacterial pathogenicity

The pathogenic potential of a bacterial strain is a multifaceted trait dependent upon the complement of core genome mutations and accessory genes present in that strain. BGWAS has identified bacterial genes linked to several diseases. These studies have been conducted on a variety of clinically important pathogens, such as *Escherichia coli* (24), *Neisseria meningitidis* (42), *Streptococcus pneumoniae* (43), *Mycobacterium tuberculosis* (44), *Helicobacter pylori* (45), *Vibrio vulnificus* (46), *Staphylococcus aureus* (47), *Klebsiella pneumoniae* (48), *Streptococcus suis* (49), *Listeria monocytogenes* (50), and *Pseudomonas aeruginosa* (51) (Table 1; Table S1).

In *Escherichia coli*, BGWAS have substantiated the *pap*GII gene as a major risk factor for urosepsis (52). Similarly, Chaguza et al. (53) identified genetic variation within non-coding and coding regions, particularly the capsule biosynthesis locus, statistically associated with neonatal group B *Streptococcus* disease onset time and meningeal invasion. In drug-sensitive *Mycobacterium tuberculosis*, 14 non-synonymous mutations across 13 genes, notably the G559D mutation in the virulence gene *ots*B1, were associated with poor treatment outcomes in tuberculosis patients (54). Although BGWAS has been utilized to identify pathogenicity-associated genetic variations in various pathogenic bacteria, most of these significant genetic variants remain experimentally unvalidated. This lack of validation leads to uncertainties regarding their actual biological functions, thereby limiting their application in disease mechanism analysis and clinical practice. Furthermore, differences in sample selection, analytical methods, and statistical criteria among studies pose challenges to the reproducibility and robustness of BGWAS findings. Therefore, future research should integrate multiomics data (e.g., transcriptomics, proteomics, and epigenomics) to enhance analytical resolution and incorporate functional experiments to validate the specific roles of key genetic variations in pathogenesis. Additionally, the introduction of machine learning and artificial intelligence techniques may further optimize BGWAS methodologies, improving the efficiency of identifying pathogenicity-associated genetic variants.

### GWAS and bacterial host specificity

Certain genetic factors in bacteria determine their capacity to infect specific hosts or exhibit a preference for particular hosts. Based on this characteristic, genetic variants associated with host specificity have been identified through GWAS in various bacteria, including *Campylobacter* (16), *Vibrio parahaemolyticus* (55), *Staphylococcus pseudintermedius* (56), *Serratia marcescens* (57), *Salmonella* (58), and *Escherichia coli* (59). In *Campylobacter*, three genes encoding vitamin B5 biosynthesis were almost universally present in cattle but were frequently absent in isolates from chickens and wild birds (16). In *Salmonella enterica* subsp. e*nterica*, BGWAS identified 52 genomic signatures that were significantly associated with isolates from avian, bovine, swine, and fish sources (58). These findings highlight the power of BGWAS in identifying genetic determinants associated with host specificity, offering valuable insights into bacterial adaptation and evolutionary trajectories.

### GWAS and other bacterial phenotypes

LABs are extensively utilized in the food industry owing to their unique carbohydrate fermentation capabilities. Consequently, elucidating the genetic mechanisms underlying the fermentation type and carbohydrate utilization in LAB is essential for optimizing their industrial processes. BGWAS has revealed that obligately heterofermentative species lack 1-phosphofructokinase, required for D-mannose degradation in the homofermentative pathway (22). In *Lactobacillus delbrueckii* subsp. *bulgaricus*, l-lactate dehydrogenase was significantly associated with the bacterial acid production rate, one of the critical fermentation characteristics (26). Moreover, the fermentation capability of *Lactococcus lactis* subsp. *lactis* was significantly associated with polymorphic loci within the *pep*F and *coi*A genes (60). These insights not only deepen our understanding of fermentation characteristics in LAB but also pave the way for precision breeding and synthetic biology applications in the dairy and fermentation industries.

Bacterial biofilms are complex microbial communities encased in extracellular polymeric substances. They facilitate bacterial colonization and significantly enhance resistance to antimicrobial agents and host immune responses. Given the clinical and environmental implications of biofilm-related persistence and resilience, uncovering the genetic basis of biofilm formation has become a key research focus. GWAS have revealed genetic variations that influence biofilm formation in various bacteria including *Pseudomonas aeruginosa* (61), *Listeria monocytogenes* (62), and *Staphylococcus saprophyticus* (63). Using GWAS and pan-GWAS, Redfern et al. (61) discovered a number of accessory genes and core-genome single nucleotide polymorphisms (SNPs) that were associated with enhanced early biofilm formation at 22°C compared to 37°C. Notably, these included a 165 kb genomic island containing multiple heavy metal resistance genes, transcriptional regulators, and methyltransferases. In *Listeria monocytogenes*, Monteith et al. (62) identified 273 genes significantly associated with enhanced biofilm-forming capacity. Further work, e.g., gene-knockout studies is required to confirm their relevance.

## THE PROCESS OF BGWAS ANALYSIS

**Fig 1:**
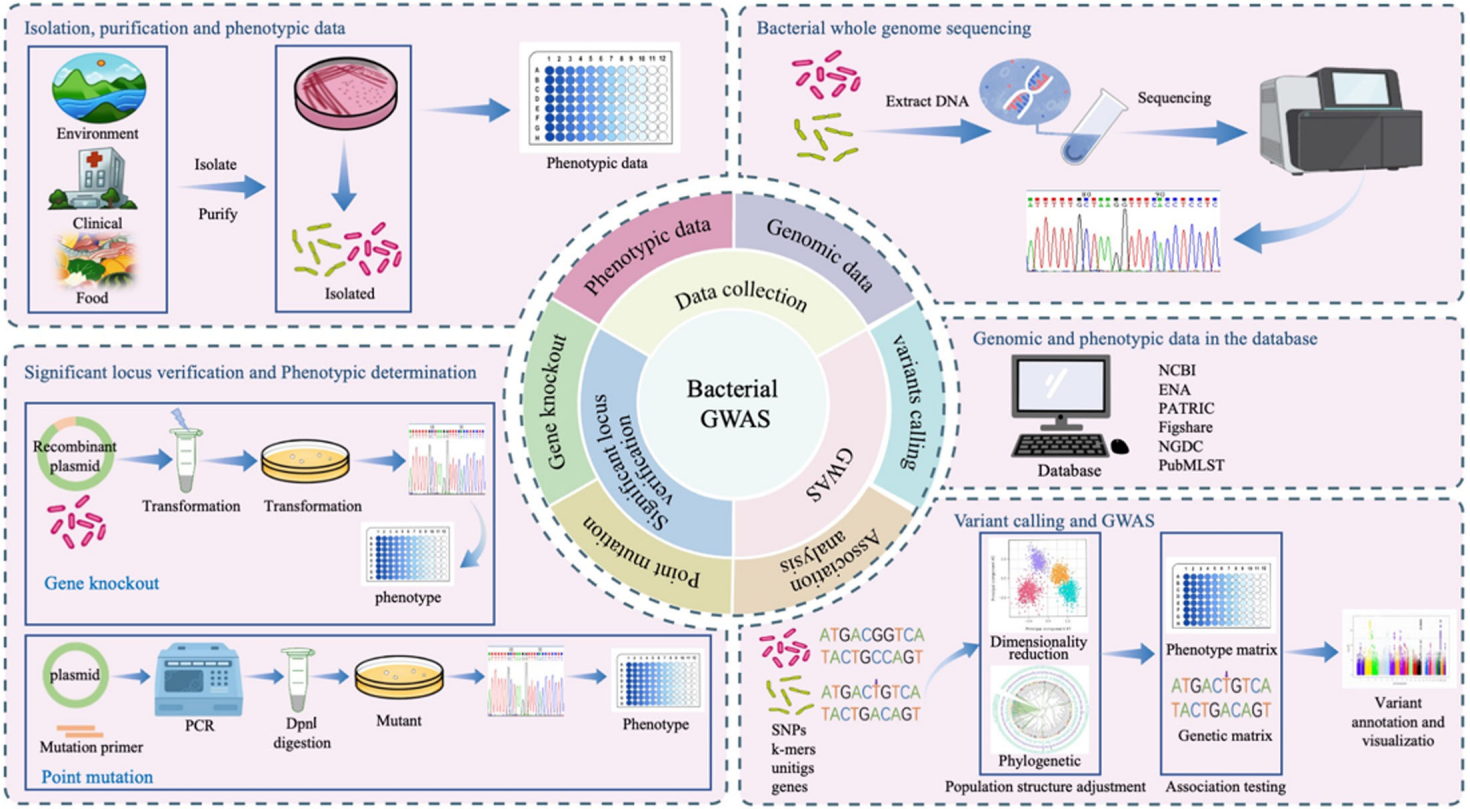
Schematic of the bacterial genome-wide association study for exploring the genetic variation underlying bacterial phenotypes. ENA, European Nucleotide Archive; NCBI, National Center for Biotechnology Information; PATRIC, Pathosystems Resource Integration Center.

**Fig 2:**
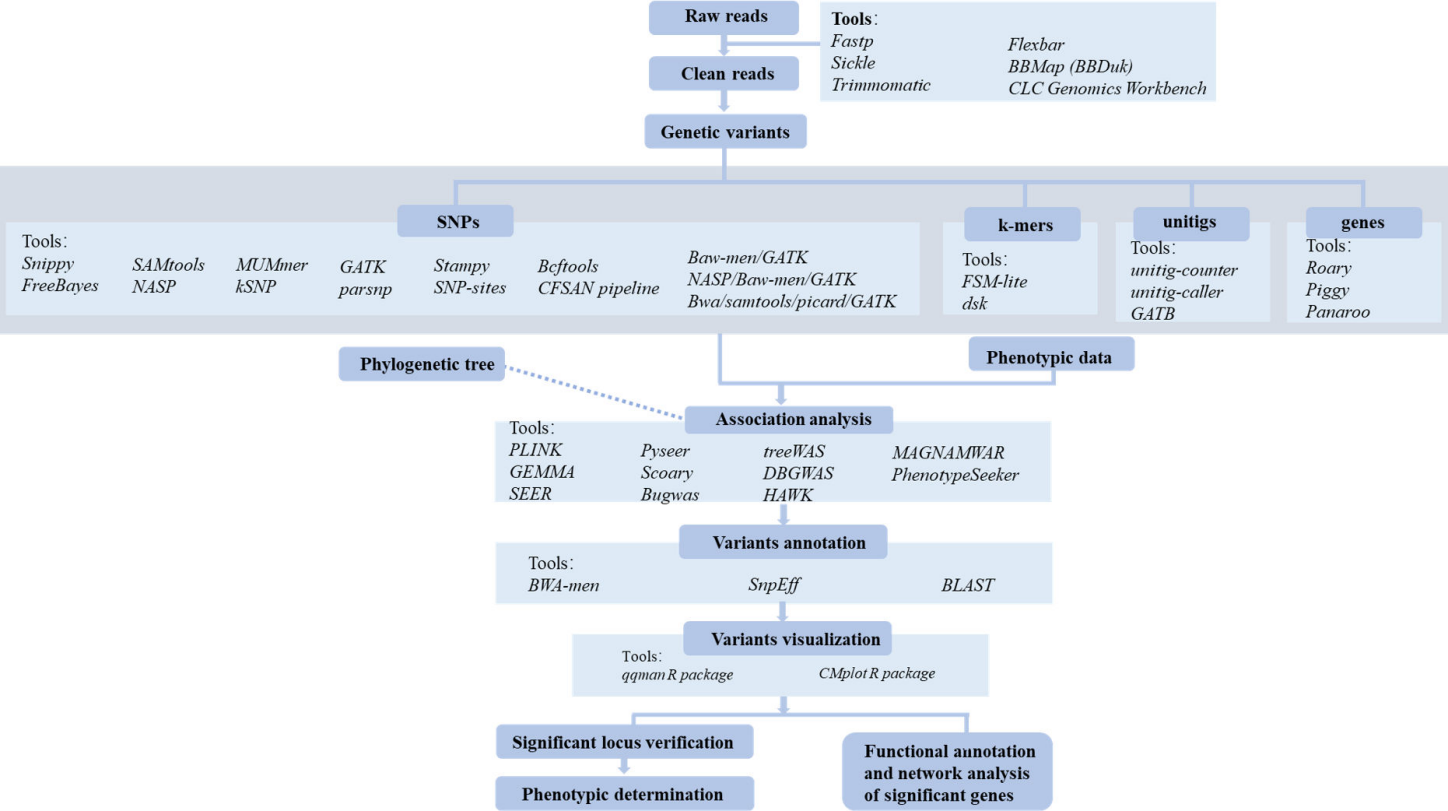
Bacterial genome-wide association study pipeline and related analysis tools.

**Fig 3:**
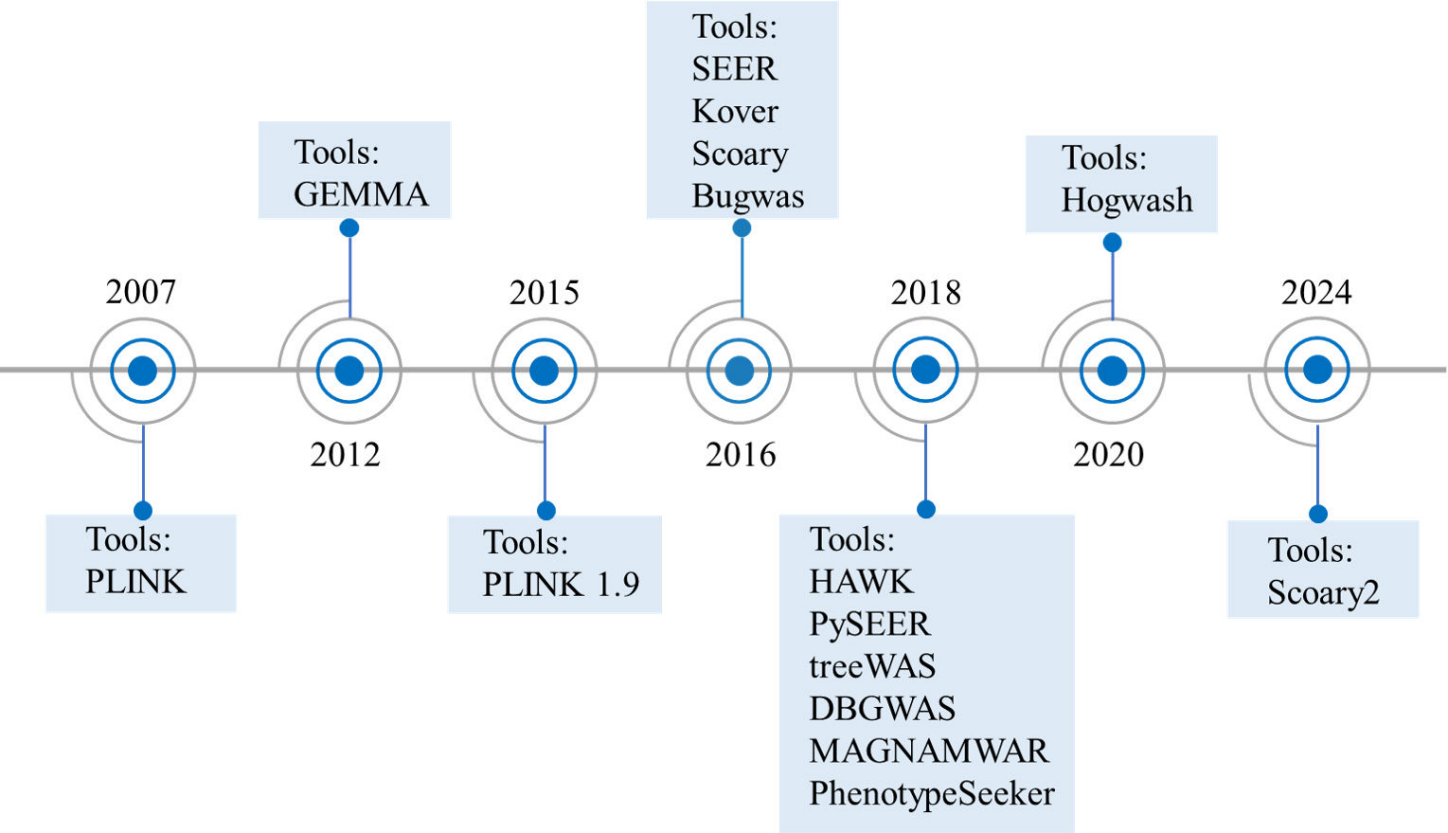
Timeline of available bacterial genome-wide association study tools.

The key steps in BGWAS include phenotype selection and measurement, genotype data acquisition, association analysis between phenotypes and genotypes, and validation of significant loci (Fig. 1 and 2). Phenotypic traits should be clearly distinguishable and readily measurable to ensure accurate association analysis. Depending on the nature of the trait and the statistical model used, both binary and continuous phenotypic data can be utilized in BGWAS. In general, bacterial genomic data are obtained through whole-genome sequencing or retrieved from public databases, such as the National Center for Biotechnology Information, the European Nucleotide Archive, and the Pathosystems Resource Integration Center. Notably, high-quality, complete genomes or well-assembled draft genomes typically offer more accurate representations of gene presence/absence, structural variants, and intergenic regions, which are crucial for detecting meaningful associations. In contrast, fragmented assemblies may lead to incomplete or erroneous detection of genetic features, potentially introducing noise or bias in association results. The types of genomic data used in BGWAS include SNPs, insertions and deletions (indels), k-mers, unitigs, and gene presence/absence patterns (64). Subsequently, various BGWAS tools can be employed to perform association analyses between phenotypic traits and genomic variants, enabling the identification of genetic factors underlying bacterial phenotypes (Fig. 3). The results are commonly visualized using Manhattan and Q-Q plots, typically generated in R with packages such as qqman or CMplot. Finally, significant loci can be validated through wet-lab experiments such as site-directed mutagenesis and gene knockout. In addition, functional annotation and network analysis of these loci can be performed to further elucidate their biological roles.

Currently, k-mers or unitigs are recommended as genomic markers for BGWAS. Using k-mers as markers allows the detection of almost any type of structural variant, including insertions, deletions, or transpositions in addition to classical SNPs (65). In contrast to SNP- or gene-based methods, k-mer analyses do not require a reference genome and can even be performed without assembling the genome sequences. Therefore, k-mer-based GWAS provide a more comprehensive approach for analyzing the association between genomic variation and phenotypic traits. However, interpreting the results of k-mer-based GWAS is challenging due to mapping difficulties and high redundancy (66). To address these limitations, unitigs remove redundancy from k-mers by collapsing all nodes representing the same sequence into a single node and branching nodes to show sequence variation (64, 66). Furthermore, Sommer et al. (67) developed a simple computational method (panfeed) that explicitly links each k-mer to their gene cluster at base-resolution level. This approach mitigates biases introduced by a global de Bruijn graph and facilitates a more accurate mapping and annotation of associated variants.

## TOOLS FOR BGWAS ANALYSIS

A variety of GWAS tools have been developed to accommodate the unique genomic characteristics of bacteria (64) (Fig. 3; Table 2). Software specifically designed for BGWAS is expected to significantly accelerate progress in this field. These tools can be broadly classified into the following categories: (i) phylogeny-based approaches can identify significant correlations between genomic variations and phenotypic traits, such as treeWAS and hogwash (68, 69); (ii) statistical analysis-based tools employ various statistical methods (e.g., *χ*^2^ test, Fisher’s exact test, Cochran–Mantel–Haenszel test, logistic regression, and linear mixed models [LMMs]) to identify potential genetic markers associated with interested phenotypes (70); (iii) machine learning techniques can reveal complex relationships between genomic features and phenotypic traits and are increasingly applied to predict bacterial phenotypic characteristics. Different BGWAS tools possess distinct advantages and limitations (64). Therefore, the selection of a suitable BGWAS approach requires careful consideration of factors such as sample size, the types of genetic variation, and the specific phenotypic traits under investigation. Moreover, integrating multiple analytical methods can further improve the efficiency and accuracy of BGWAS.

**TABLE 2:** Overview of commonly used tools for BGWAS^*a*^

Tools	Variants	Phenotype	Association analysis	Population structure correction	Multiple testing correction	Comment
PLINK	SNPs and genes	Binary and continuous phenotype	The standard case/control allelic test; Cochran–Armitage trend test; Fisher’s exact test; genotypic tests (general, dominant, and recessive models); and Cochran–Mantel–Haenszel tests	Cluster individuals into homogeneous subsets; perform classical MDS; identify outlying individuals; and complete linkage hierarchical clustering	Bonferroni, Sidak, FDR, etc.	Efficiently processes and analyzes large-scale genotype data; a powerful, user-friendly tool; not developed specifically for BGWAS
GEMMA	SNPs, k-mers, unitigs, and genes	Binary and continuous phenotype	The univariate LMM and the multivariate linear mixed model	Random effects in LMM	–^*b*^	A software toolkit for fast application of LMMs and related models to GWAS, computationally practical for large numbers of individuals
Bugwas	SNPs, k-mers, unitigs, and genes	Binary and continuous phenotype	Logistic regression implemented in R (SNPs or genes), *χ*^2^ test implemented in C++ (k-mers), and LMM	Principal components and LMM	Bonferroni	R package to test for locus and lineage associations in bacterial GWAS
SEER	SNPs, k-mers, unitigs, and genes	Binary and continuous phenotype	Logistic regression, Firth regression, and linear regression	MDS based on k-mer distance matrix	Bonferroni	Supports *de novo* assembled contigs or raw read data as input and superseded by Pyseer
Pyseer	SNPs, k-mers, unitigs, and genes	Binary and continuous phenotype	The fixed effect generalized linear regression model, an LMM, and FaST-LMM	MDS based on distance matrix, random effects to correct for population structure (“-lmm” mode) by providing a similarity matrix acquired from the core genome phylogeny	Bonferroni, FDR, Holm, and Hommel	Supports multiple variant types from VCF/Rtab and auto label matching
treeWAS	SNPs, k-mers, unitigs, and genes	Binary phenotype data and discrete interval (categorical) and continuous phenotypic data	The “terminal score,” the “simultaneous score,” and the “subsequent score”	Phylogenetic approach	Bonferroni correction (default) and false discovery rate	R package for microbial GWAS using phylogeny-based approach and superior sensitivity and specificity compared to cluster-based and dimension-reduction methods
MAGNAMWAR	Orthologous genes	Binary and continuous phenotypes	Mixed models, survival models, and Wilcoxon tests	PCA	–^*b*^	R package for GWAS of bacterial orthologs, supports orthologs derived from bacterial genomes or metagenomes
HAWK	k-mers	Categorical phenotype and quantitative phenotypes	A likelihood ratio test for nested models, logistic regression (categorical phenotype), and linear regression (quantitative phenotypes)	Perform PCA on k-mer present/absent matrix	Bonferroni correction and Benjamini–Hochberg correction	Accepts raw FASTQ files as input
DBGWAS	k-mers and unitigs	Binary phenotypes	Uses Bugwas for identifying significant associations between unitigs and phenotypes	LMM	Benjamini–Hochberg	Alignment-free method requiring only contigs and phenotypes, provides a graphical framework to help interpret GWAS results, and computationally efficient and user-friendly
Hogwash	SNPs, k-mers, and genes	Binary and continuous phenotypes	Continuous test, synchronous test, and PhyC	Phylogenetic approach	False discovery rate	R package for convergence-based BGWAS algorithms
Scoary	Genes, SNPs, and k-mers	Binary phenotypes	Fisher’s exact test	Implements the pairwise comparison algorithm	Bonferroni and Benjamini–Hochberg	An easy-to-use, ultra-fast tool, correlates gene presence/absence from pangenome analysis with phenotype

^*a*^ FDR, false discovery rate; HAWK, Hitting Associations with k-mers; LMM, linear mixed model; MAGNAMWAR, Mono-Associated Gnotobiotic Animal Metagenome-Wide Association; MDS, multidimensional scaling; PCA, principal component analysis.

^*b*^ – indicates that the information was not explicitly stated in the referenced software publication.

### PLINK

In 2007, Purcell et al. (71) developed PLINK, a free and open-source C/C++ toolset for performing whole-genome association analyses. It efficiently handles large genetic data sets comprising thousands of samples. To address the challenges posed by increasingly large and complex data sets, Chang et al. (72) developed a second-generation PLINK (PLINK version 1.9), which introduces numerous algorithmic improvements, extends the data format, and offers significant enhancements in performance and compatibility compared to the first-generation version. In addition, the “epistasis” option in PLINK enables the analysis of SNP–SNP epistatic interactions (73). Notably, PLINK is originally developed for human GWAS; it cannot utilize triallelic SNPs (71). To compensate for this limitation, Weber et al. (21) replaced minor with major variants when employing PLINK in BGWAS.

PLINK has been employed to examine associations between genetic variants and phenotypes in various bacterial species, such as daptomycin and ceftaroline resistance in *Staphylococcus aureus* (21); penicillin, trimethoprim, co-trimoxazole, erythromycin, ciprofloxacin, ofloxacin, and cephalosporins resistance in *Streptococcus pneumoniae* (74); ethambutol, isoniazid, rifampicin, and streptomycin resistance in *Mycobacterium tuberculosis* (75); and pathogenicity in *Streptococcus pneumoniae* (76) and *Enterococcus faecalis* (77).

### GEMMA

In 2012, Zhou and Stephens (78) presented a new, more efficient method for exact calculations, which was implemented in the software GEMMA. Currently, GEMMA has become a software toolkit for fast application of LMMs and related models to GWAS and other large-scale data sets (https://github.com/genetics-statistics/GEMMA). Univariate linear mixed models (for single phenotype) and multivariate linear mixed models (for multiple phenotypes) have been used to test associations between genetic variants and correlated phenotypes while controlling for population stratification. GEMMA has also been applied in bacterial GWAS, such as symbiotic, metabolic, growth, and environmental tolerance traits in *Ensifer meliloti* (79); growth plasticity in *Staphylococcus aureus* (25, 80); drug resistance in *Mycobacterium tuberculosis* (20) and *Acinetobacter baumannii* (81); and pathogenicity in *Streptococcus agalactiae* (53), *Mycobacterium tuberculosis* (54), and *Neisseria meningitidis* (82).

### Scoary

In 2016, Brynildsrud et al. (83) developed Scoary (https://github.com/AdmiralenOla/Scoary), an accessible, powerful, and user-friendly tool for identifying associations between the presence or absence of pan-genome genes and observed phenotypes. This approach, commonly known as pan-GWAS, represents a distinct strategy from traditional SNP-based GWAS. Scoary requires a gene presence/absence matrix generated by Roary or Panaroo, along with a corresponding phenotype matrix file. A phylogenetic tree can be either provided by the user or calculated internally through the isolate Hamming distances of the input genotype file. In addition, the vcf2scoary tool can convert the predicted SNPs from bacterial genomes into Scoary format, which can then be used to generate a presence/absence genetic matrix file (84). Scoary typically employs Fisher’s exact test to assess associations between gene presence/absence and the phenotype of interest. To control spurious associations from stratified populations, it implements the pairwise comparison algorithm. Additionally, multiple testing is corrected using the Bonferroni and Benjamini–Hochberg adjustments. According to previous studies, Scoary is the most frequently cited software in BGWAS to date (64, 85) and is widely used for analyzing genetic variants associated with phenotypes, such as pathogenicity in *Escherichia coli* (24), *Glaesserella parasuis* (27), *Streptococcus pneumoniae* (43), *Staphylococcus aureus* (47), *Cutibacterium acnes* (86), and *Mycobacterium avium* subsp. *paratuberculosis* (87); biofilm formation in *Glaesserella parasuis* (27) and *Pseudomonas aeruginosa* (61); lineage specificity in *Brucella* spp. (84); and host specificity in *Staphylococcus pseudintermedius* (56). However, Scoary still has certain limitations: (i) it can process only a limited number of samples, making it unsuitable for analyzing large-scale genomic data sets involving thousands of isolates, and (ii) it is not designed to handle continuous traits and requires phenotypic data to be converted into binary format.

In 2024, Roder et al. (85) developed Scoary2, a software that links high-dimensional phenotypic data from omics technologies with genomic data to elucidate the impact of specific genes on phenotypes, which completely rewrote and extended Scoary.

### Bugwas

In 2016, Earle et al. (31) developed an end-to-end BGWAS pipeline named Bugwas (https://github.com/sgearle/bugwas), which can be implemented in R, Python, and C++. Bugwas is a GWAS tool tailored for bacterial population genomics, enabling the detection of genetic variants associated with phenotypic traits while accounting for population structure. It employs LMMs and principal component analysis (PCA) to control population stratification. To date, Bugwas has been utilized to analyze genetic variations associated with bacterial phenotypes, such as pathogenicity in *Helicobacter pylori* (45) and *Neisseria meningitidis* (82); delta-toxin production of *Staphylococcus aureus* (88); and drug resistance in *Acinetobacter baumannii* (81) and *Mycobacterium tuberculosis* (89).

### SEER

In 2016, Lees et al. (65) developed sequence element enrichment analysis (SEER; https://github.com/johnlees/seer), a scalable tool for BGWAS that identifies k-mer-based genetic elements associated with phenotypic traits. SEER is well suited for large-scale data sets involving thousands of bacterial genomes. It supports input from either *de novo* assembled contigs or raw sequencing data and computes k-mers across samples using one of three approaches: fsm-lite (the default method), distributed string mining, or disk streaming of k-mers. Furthermore, SEER corrects for population structure by applying multidimensional scaling (MDS) to a pairwise distance matrix derived from the k-mer profiles. It performs association testing using logistic regression for binary traits and linear regression for continuous traits. SEER has also been used to investigate the genetic basis of bacterial phenotypes, including drug resistance in *Staphylococcus aureus* (21) and delta-toxin production in *Staphylococcus aureus* (88). However, the effectiveness of SEER is constrained by the levels of recombination and convergent evolution in the studied population, since the discovery of causal sequence elements is principally constrained by the extent of linkage disequilibrium.

### Pyseer

In 2018, Lees et al. (90) developed Pyseer (https://github.com/mgalardini/pyseer), a software package designed for microbial GWAS. It is a Python-based reimplementation and extension of SEER. Compared to SEER, Pyseer is more user-friendly and easier to install via Conda. Pyseer supports genome-wide association studies using a wide range of genetic features, including SNPs, indels, k-mers, unitigs, orthologous genes, and aligned intergenic regions. This flexibility enables comprehensive analysis of both core and accessory genome variation. In addition to implementing the generalized linear model as in SEER, Pyseer also supports LMMs for association analysis. Furthermore, it includes a method to estimate possible lineage effects, based on the procedure used in Bugwas. In pyseer, several strategies are implemented to control for population stratification: (i) conducting multidimensional scaling on the pairwise distance matrix, (ii) incorporating a high-quality phylogeny to adjust for population structure in a manner analogous to phylogenetic regression, and (iii) using a kinship matrix estimated from a subset of variants or from a phylogeny as random effects in LMM. Additionally, Pyseer can automatically detect and match the sample label order of the input files.

Pyseer is widely used in BGWAS to identify genetic variants associated with various phenotypes, such as drug resistance in *Neisseria gonorrhoeae* (19), *Klebsiella pneumoniae* (32), *Escherichia coli* (35), and *Staphylococcus aureus* (91); pathogenicity in *Pseudomonas aeruginosa* (51), *Streptococcus pneumoniae* (43), *Vibrio vulnificus* (46), *Escherichia coli* (52), *Helicobacter pylori* (92), and *Staphylococcus aureus* (93); host specificity in *Escherichia coli* (59) and *Vibrio parahaemolyticus* (55); and biofilm formation in *Escherichia coli* (17), *Listeria monocytogenes* (62), and *Pseudomonas aeruginosa* (61).

### treeWAS

In 2018, Collins and Didelot (68) developed treeWAS (https://github.com/caitiecollins/treeWAS), a phylogenetic method implemented in R for performing BGWAS. TreeWAS conducts GWAS through a six-step workflow, in which genotype–phenotype associations are evaluated using three scores: the terminal score, the simultaneous score, and the subsequent score. Notably, treeWAS effectively corrects for the confounding effects of clonal population stratification and homologous recombination and is therefore applicable to organisms ranging from purely clonal to frequently recombining. Additionally, phylogenetic trees used in treeWAS are recommended to be constructed using ClonalFrameML (https://github.com/xavierdidelot/ClonalFrameML), a software package specifically designed to infer recombination in bacterial genomes (94). TreeWAS has been employed to investigate genetic variations underlying a variety of bacterial phenotypes, such as pathogenicity in *Listeria monocytogenes* (50), *Neisseria meningitidis* (42), *Streptococcus suis* (49), and *Mycobacterium avium* subsp. *paratuberculosis* (87); drug resistance in *Klebsiella pneumoniae* (41) and *Gallibacterium anatis* (95); and fermentation-related traits in *Lactobacillus delbrueckii* subsp. *bulgaricus* (26) and *Lactococcus lactis* subsp. *lactis* (60).

### MAGNAMWAR

MAGNAMWAR (mono-associated gnotobiotic animals metagenome-wide association) is an R package designed for GWAS of bacterial orthologs. Initially, OrthoMCL is utilized to identify bacterial orthologous genes, followed by MAGNAMWAR to conduct various statistical tests (mixed models, survival models, and Wilcoxon tests) to analyze correlations between genes and phenotypes. MAGNAMWAR accounts for population stratification using principal component analysis. However, it is specifically limited to the analysis of gene presence/absence data and does not support the assessment of other types of genetic variation (96).

### HAWK

HAWK (hitting associations with k-mers), implemented in C++, performs likelihood testing of k-mer counts to detect association with a given trait and applies logistic regression to account for confounding effects. Initially, HAWK extracts k-mers from sequencing data, calculates their frequencies in each sample, and then applies a statistical model to evaluate the association between individual k-mer frequencies and the trait. Finally, HAWK integrates all significant k-mers to identify genomic regions likely associated with the trait. Additionally, HAWK employs PCA to detect and correct for population stratification (97, 98). It has been applied to investigate ampicillin resistance in *Escherichia coli* (98).

### DBGWAS

DBGWAS (https://gitlab.com/leoisl/dbgwas) is a k-mer-based BGWAS tool that utilizes compressed De Bruijn graphs (DBGs) to produce interpretable genetic variants associated with distinct phenotypes (66). DBGWAS initially constructs a DBG from the input draft genome data using GATB and then compresses it into a compressed De Bruijn graph (cDBG) via a graph traversal algorithm. An LMM, implemented in the Bugwas, is then used to assess the association between cDBG nodes (unitigs) and the phenotype of interest, and the associated nodes are remapped onto the cDBG. DBGWAS provides a web-based interface that enables users to further explore the results through interactive visualizations (66). To date, DBGWAS has been employed to investigate genetic variants associated with various bacterial phenotypes, such as pathogenicity in *Vibrio vulnificus* (46) and *Staphylococcus aureus* (99); drug resistance in *Mycobacterium tuberculosis* (89), *Mycoplasma bovis* (100), and *Achromobacter* spp. (101); and natural transformation in *Legionella pneumophila* (102).

### Hogwash

Hogwash (https://github.com/katiesaund/hogwash) is an open-source R package that implements three algorithms for convergence-based BGWAS (69). The three algorithms include the Synchronous Test, the Continuous Test, and PhyC. Among them, PhyC is an independent test that leverages evolutionary convergence. The Synchronous Test, which is similar to the “simultaneous score” in treeWAS, is a strict variant of PhyC that requires a closer relationship between genotype and phenotype. PhyC and the Synchronous Test are applicable only to binary phenotypes, whereas the Continuous Test is a convergence-based BGWAS approach designed to analyze continuous phenotypes. Hogwash mitigates the multiple testing burden by focusing only on genotype–phenotype pairs with detectable convergence and employs false discovery rate correction rather than the more conservative Bonferroni adjustment. However, Hogwash requires genetic variations to occur across multiple different lineages, which may restrict its applicability. Its computational demands also increase with data set size, potentially limiting its scalability to larger genomic studies (69). According to previous studies, Hogwash has been applied to investigate genetic variants associated with pathogenicity in *Clostridioides difficile* (103) and drug resistance in *Mycobacterium tuberculosis* (104).

### Machine learning

Kover (https://github.com/aldro61/kover) is a machine learning framework that creates models of genotype–phenotype relationships based on k-mers. It employs the Set Covering Machine to identify k-mers significantly associated with the phenotype of interest (105, 106).

PhenotypeSeeker is a machine learning tool composed of “PhenotypeSeeker modeling” and “PhenotypeSeeker prediction” that identifies phenotype-specific k-mers, generates phenotype prediction models, and predicts phenotypes based on bacterial sequencing data. PhenotypeSeeker is an open-source tool implemented in Python. It accepts genotypic input in the form of either assembled contigs or raw sequencing reads and supports both binary and continuous phenotypic data. All k-mer-related operations are performed using the GenomeTester4 package. Clonal population structure is corrected through a sequence weighting approach that reduces the weight of isolates with closely related genomes. PhenotypeSeeker uses the *χ*^2^ test and logistic regression for binary traits and the Welch two-sample *t*-test with linear regression for continuous traits (107). It has been applied to investigate genetic variants associated with virulence in *Klebsiella pneumoniae*, ciprofloxacin resistance in *Pseudomonas aeruginosa*, and azithromycin resistance in *Clostridioides difficile* (107).

## BGWAS CONFOUNDING FACTORS

In BGWAS, correcting for population structure is crucial to avoid spurious associations, which may arise from the non-random distribution of the sample rather than genuine genotype–phenotype correlations (23). Several approaches have been used to correct for the effects of population structure (64):

1. Dimensionality reduction techniques (i.e., PCA and MDS) are employed to capture genetic variation attributable to population structure (31, 90). By projecting the data onto a few principal components that explain the majority of the variation, these components can be included as covariates in association models to adjust for population structure. For example, Pyseer and SEER can correct for clonal population structure by performing MDS on a distance matrix (65, 90). In addition, PLINK, MAGNAMWAR, HAWK, and Bugwas employ PCA to correct for bacterial population structure (66, 71, 96, 98).
2. Phylogeny-based correction methods account for the evolutionary relationships among isolates to effectively control population structure biases caused by lineage relatedness, thereby improving the accuracy of association analyses.
3. Random effects were included in the mixed models to account for genetic relatedness among strains. In LMM and FaST-LMM, a kinship matrix derived from core genome phylogeny is used to correct for population structure (52, 90).
4. Strategically stratifying or clustering strains and subsequently conducting association testing within these subgroups can mitigate the impact of population structure on overall analysis results. According to previous studies, HierBAPs and FastStructure software can be used to construct population stratifications (25, 26). Nevertheless, effectively controlling for population structure in BGWAS remains a major challenge. Additionally, correcting for population stratification significantly reduces the power of GWAS.

LD is also a crucial factor that must be considered in BGWAS (23). It refers to the non-random association of alleles at different loci within individuals in a given population (108). Due to LD, non-causal variants may co-occur with causal variants that genuinely influence a specific phenotype. Therefore, reducing LD-induced false positives is essential for improving the reliability of BGWAS results. Correcting for bacterial population stratification can also reduce false positives caused by LD. In addition, conditional GWAS can effectively reduce false-positive associations caused by LD. Ma et al. (19) conducted a GWAS conditioned on known resistance mutations to mitigate linkage-mediated confounding. In addition, expanding the number of strains can mitigate the impact of LD.

Multiple testing inevitably leads to false positives in GWAS results. GWAS performs a separate association test for each genotype and phenotype of interest, and each test has a certain probability of false positives. When multiple independent hypothesis tests are performed, the overall false positive rate can increase significantly, even if the false positive rate of each individual test is low. To address false positives resulting from multiple testing, statistical corrections such as the Bonferroni and Benjamini–Hochberg methods are commonly used (24, 64). Tools like PLINK, Bugwas, SEER, and Pyseer implement the Bonferroni correction, while treeWAS and DBGWAS employ the Benjamini–Hochberg correction (Table 2). Additionally, reducing the number of tests can help alleviate the burden of multiple testing.

## CONCLUSION

BGWAS introduce new methodologies and opportunities for analyzing the genetic variations underlying bacterial phenotypes. Unlike traditional bacterial genome analysis techniques, GWAS examine phenotypic mechanisms at the population level, enabling a more comprehensive exploration of potential genetic determinants. Furthermore, software tailored for BGWAS has been developed to suit the unique characteristics of bacterial genomes, enhancing its application in bacteriology and improving the precision of analytical results. Nonetheless, confounding factors such as population structure, LD, and multiple testing continue to influence the outcome of BGWAS and must be considered during analysis. Although GWAS has some applications in revealing the virulence, pathogenicity, and drug resistance mechanisms of foodborne pathogens, compared to its use in plant phenotype coding mechanisms and disease etiology exploration, the usage of this method in foodborne pathogens is still relatively limited. The application in probiotics is even less so. Therefore, it is necessary to strengthen the application of GWAS in the study of phenotype mechanisms related to foodborne pathogens and probiotics because of its powerful analysis ability. In the future, BGWAS can be used to investigate genetic variations in a broader array of bacterial species. Additionally, enhancing the sharing of bacterial phenotypic and genomic data is essential.

## SUPPLEMENTAL MATERIAL

Table S1 aem.02512-24-s0001.xlsx Articles published on PubMed and Web of Science.

ASM does not own the copyrights to Supplemental Material that may be linked to, or accessed through, an article. The authors have granted ASM a non-exclusive, world-wide license to publish the Supplemental Material files. Please contact the corresponding author directly for reuse.
